# Polyacrylonitrile/Silver Nanoparticles Composite for Catalytic Dye Reduction and Real-Time Monitoring

**DOI:** 10.3390/polym17131762

**Published:** 2025-06-26

**Authors:** Christian Narváez-Muñoz, Sebastián Ponce, Carlos Durán, Cristina Aguayo, Cesar Portero, Joseph Guamán, Alexis Debut, Magaly Granda, Frank Alexis, Ezequiel Zamora-Ledezma, Camilo Zamora-Ledezma

**Affiliations:** 1 Departamento de Ciencias de la Energía y Mecánica, Universidad de las Fuerzas Armadas—ESPE, Sangolquí 171103, Ecuador; caduran1@espe.edu.ec (C.D.); caportero@espe.edu.ec (C.P.); 2 Centre Internacional de Mètodes Numérics en Enginyeria (CIMNE), C/Gran Capitán s/n, Campus Nord, 08034 Barcelona, Spain; 3 Departamento de Ingeniería Química, Colegio de Ciencias e Ingenierías, Universidad San Francisco de Quito (USFQ), Quito 170901, Ecuador; sponce@usfq.edu.ec; 4 Universidad de las Fuerzas Armadas—ESPE, Sangolquí 171103, Ecuadorjapdebut@espe.edu.ec (J.G.); aguaman19@espe.edu.ec (A.D.); mggranda2@espe.edu.ec (M.G.); 5 Departamento de Ingeniería Química, Colegio de Ciencias e Ingeniería, Instituto de Energía y Materiales, Instituto de Microbiología, Universidad San Francisco de Quito (USFQ), Quito 170901, Ecuador; falexis@usfq.edu.ec; 6 Laboratorio de Agroecosistemas y Cambio Climático—FAGROCLIM, Departamento de Ciencias Agrícolas, Facultad de Ingeniería Agrícola, Universidad Técnica de Manabí—UTM, Lodana 13132, Ecuador; ezequiel.zamora@utm.edu.ec; 7 Bioengineering & Regenerative Medicine Research Group (Bio-ReM), Escuela de Ingeniería, Arquitectura y Diseño (EIAD), Universidad Alfonso X el Sabio (UAX), Avenida de la Universidad 1, Villanueva de la Cañada, 28691 Madrid, Spain

**Keywords:** polyacrylonitrile nanofibers, silver nanoparticle, catalytic dye reduction, green synthesis, real-time monitoring, sensors, 3D printing, hierarchical structures, electrospun fiber

## Abstract

This study presents a one-step electrospinning method to fabricate polyacrylonitrile (PAN) nanofibers embedded with green-synthesized silver nanoparticles (AgNPs) for efficient catalytic dye reduction and real-time monitoring. Utilizing avocado seed extract for AgNP synthesis, the resulting composite nanofibers exhibit uniform nanoparticle dispersion and enhanced surface area, significantly improving adsorption and catalytic properties. The membranes demonstrated outstanding catalytic activity, achieving over 95% degradation of methyl orange within 45 min when paired with sodium borohydride, and maintained structural integrity and performance over ten reuse cycles. The integration of a novel 3D-printed support enabled scalability, allowing a 60-fold increase in treatment volume without compromising efficiency. Additionally, the composite’s electrical conductivity changes enabled the real-time monitoring of the dye reduction process, highlighting its dual functionality as both catalyst and sensor. These results encourage the potential of PAN/AgNPs supported on a 3D-printed structure nanofiber membranes for scalable, sustainable wastewater treatment and in situ reaction monitoring.

## 1. Introduction

Synthetic dyes in wastewater present severe environmental challenges due to their persistence and potential carcinogenic effects [1,2,3]. Traditional treatment methods for dye removal are often hindered by high costs, complex processes, and secondary pollution [4,5]. In contrast, adsorption and catalysis offer promising alternatives that combine simplicity, efficiency, and cost-effectiveness. Integrating adsorbent materials with catalytic functionality is particularly advantageous [6,7,8]. Among various catalytic agents, noble metal nanoparticles, especially silver nanoparticles (AgNPs), have attracted considerable attention due to their catalytic properties [9,10]. AgNPs exhibit desirable characteristics such as conductivity, stability, and even antibacterial effects [11]. Despite these advantages, the widespread practical implementation of AgNPs in wastewater treatment systems faces several critical limitations. Firstly, their high intrinsic surface energy inevitably promotes their spontaneous and often irreversible aggregation in aqueous suspensions [12]. This agglomeration significantly diminishes their accessible surface area, consequently leading to a substantial reduction in catalytic efficiency and overall operational stability. Secondly, the nanoscale dimensions of AgNPs present formidable challenges in terms of their facile separation and recovery from treated water, often necessitating energy-intensive and costly separation techniques such as centrifugation or membrane filtration, thereby hindering their economic viability and reusability in large-scale applications. Furthermore, the potential uncontrolled release of AgNPs into the environment raises considerable ecotoxicological concerns for communities within wastewater treatment infrastructure [13]. Lastly, while traditional chemical synthesis routes for AgNPs are well-established, they frequently involve the use of hazardous and expensive chemical precursors and reducing agents, posing environmental and economic challenges for scalable production [14]. Although green synthesis approaches leveraging bioactive compounds from readily available biological sources like plant extracts offer a more sustainable and environmentally benign pathway for AgNP fabrication [15,16], these methods do not inherently resolve the post-synthesis issues of aggregation and recovery associated with bare nanoparticles.

One of the most effective strategies to address NP aggregation is the incorporation of NPs into high-surface-area matrices [17,18]. Such composite materials provide a stable platform that not only mitigates nanoparticle aggregation by offering physical confinement and chemical anchoring sites. Moreover, the supporting matrix can contribute synergistic properties, such as enhanced adsorption capacity or improved mechanical stability, further augmenting the overall performance of the catalytic system. In this context, ElectroHydroDynamics (EHD)-based manufacturing processes, particularly electrospinning, offer an exceptionally versatile and scalable technique for fabricating polymer fiber mats [19]. Electrospinning harnesses electrostatic forces to draw polymer solutions or melts into ultrafine fibers, enabling precise control over fiber diameter, morphology, and surface characteristics, leading to the creation of nanofiber architectures [20]. Electrospun nanofiber mats (EFMs) intrinsically possess a high surface-area-to-volume ratio, extensive porous networks, and customizable surface functionalities, rendering them outstanding candidates for achieving uniform nanoparticle dispersion and demonstrating superior performance in catalytic [21,22] and sensing applications [23].

Various methods have been employed to integrate nanoparticles into EFMs, such as surface treatment [24], in situ reduction [25,26], hydrothermal treatment [27], and calcination [28]. For example, AgNP-embedded polyvinyl alcohol (PVA) fibers have been synthesized by electrospinning a PVA/AgNO_3_ solution and subsequent thermal treatment [29]. Alternatively, AgNPs have been incorporated into chitosan/gelatin nanofibers using reducing agents like microcrystalline chitosan [30] or chitosan oligosaccharides [31,32]. AgNPs have also been deposited onto cross-linked PVA fibers by immersing them in an AgNO_3_ solution [33]. While these methods are effective, they often involve complex and time-consuming multi-step processes. To simplify the process, single-step electrospinning offers a straightforward method for fabricating NP-embedded EFMs [34]. This technique enables the simultaneous formation of NPs and fibers by incorporating NP precursors directly into the polymer solution. For instance, researchers have produced polymer/AgNP composite nanofibers in a single step by utilizing the polymer solution as a reducing agent [35,36,37]. Alternatively, pre-synthesized AgNPs can be incorporated into the electrospinning solution to create composite fibers [38]. A diverse range of polymeric systems have been employed in the fabrication of AgNP-embedded EFMs, including polyacrylonitrile (PAN) [39], nylon [40], poly(vinyl pyrrolidone) (PVP) [41], and poly(vinylidene fluoride) (PVDF) [42], among others.

**Scheme 1:**
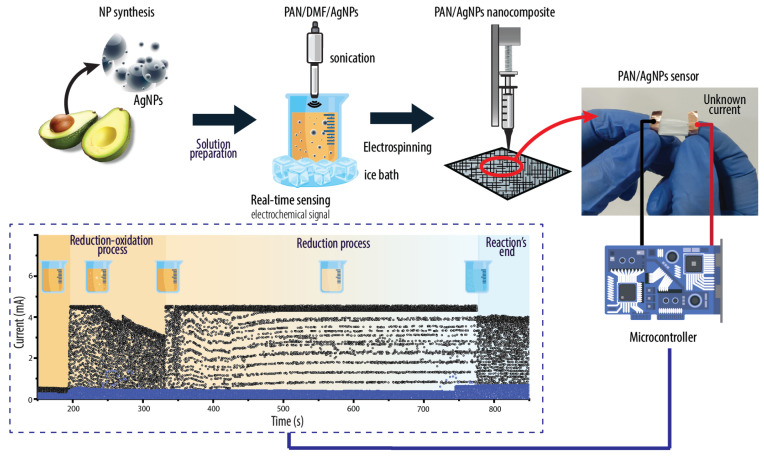
Schematic illustration of the synthesis of PAN/AgPNs nanocomposite.

Despite significant advancements, most prior research has focused on isolated aspects of the properties or applications of NP-embedded EFMs. However, there is a growing need for multifunctional materials that combine catalytic activity with sensing capabilities. Such materials can enable the real-time monitoring of analyte interactions and the dynamic control of chemical reactions. Some studies have explored the semiconductor properties of nanoparticles for advanced sensor platforms, such as those based on Raman spectroscopy [43]. However, the high cost and specialized expertise required for Raman spectroscopy limit its widespread applicability. This work presents a one-step electrospinning process to fabricate polyacrylonitrile nanofibers embedded with green-synthesized AgNPs. The resulting functional composite nanofibers offer (as illustrated in the Scheme 1) a platform for both catalytic applications and local sensing. To evaluate the catalytic activity of the composite nanofibers, we used the reduction of methyl orange by sodium borohydride as a model reaction. This thermodynamically favorable reaction, accompanied by a distinct color change from orange to colorless, was monitored using UV-vis spectroscopy. While UV-vis spectroscopy provides valuable insights into the overall reaction progression, it is an ex situ technique, meaning samples must be periodically withdrawn from the reaction mixture for analysis. This batch sampling approach offers only discrete snapshots of the reaction, limiting the ability to capture rapid kinetic changes or the precise moment of catalyst deactivation. To overcome these limitations and unlock a deeper understanding of the catalytic mechanism and reaction dynamics, the development of real-time, in situ monitoring capabilities is indispensable. Therefore, by leveraging the electrical properties of AgNPs, we integrated the composite nanofibers into a sensing platform to track the kinetics of chemical reactions. This platform enables continuous monitoring, even in challenging environments, such as dye reduction processes. As the chemical reaction proceeds, it induces changes in the electrical current of the AgNP/PAN nanofibers. By measuring these current changes, we can obtain real-time information about the reaction kinetics, providing valuable insights into the catalytic mechanism and reaction rate.

## 2. Materials and Methods

### 2.1. Materials

This research employed commercially available polyacrylonitrile K150 (LookChem, Shanghai, China) and dimethylformamide (DMF) (99.5% purity, 0.945 g/mL density, 73.09 g/mol molecular weight) from Fisher Scientific (Cali, Colombia). Reagent-grade silver nitrate salt (AgNO_3_) from Scharlau was used for silver nanoparticle synthesis, along with distilled water. Avocado (*Persea americana*) seeds were procured from local Quito markets and restaurants.

### 2.2. Preparation of AgNPs

Extract preparation from avocado seed residues followed a standardized method developed in a previous contribution [16]. Avocado seeds were first processed and then placed into a Soxhlet extractor. Around 9 g of avocado seeds were placed in a thimble, and 150 mL of distilled water was introduced into the distillation flask. The extraction process lasted for 4 h. Following this, the aqueous extract was concentrated using a rotary evaporator and subsequently freeze-dried to yield the final extract. For NP synthesis, 300 mg of dried extract and 500 mg of AgNO_3_ were mixed in 20 mL and stirred in dark conditions for 30 min. Then, the solution was placed in a test tube and illuminated with a commercial blue LED floodlight (100 W, IP66) at room temperature for four hours. The resulting colloidal suspension was isolated by centrifugation at 6000 rpm for 15 min. The solid precipitate was collected and dried overnight at 60 °C.

### 2.3. Preparation of Electrospun PAN/AgNPs Membranes

Electrospun nanocomposites were fabricated based on our previously established methodology [44], which utilized the Taguchi method and morphological analysis to define an optimal phase diagram for fiber production. As per the established recommendations, PAN was dissolved in DMF at a concentration of 12 wt.%. The PAN/DMF solution was prepared by first adding 50 mL of DMF to a 100 mL flask, followed by the gradual addition of a small amount of PAN to prevent polymer clumping. Once complete dissolution was achieved, the remaining PAN was added to obtain a uniform white solution. The mixture was stirred magnetically at 400 rpm for 2 h at room temperature. The flask was sealed throughout the stirring process to minimize solvent evaporation. Subsequently, different amounts of AgNPs powder (2.5, 5, and 7.5 wt.%) were directly incorporated into the PAN/DMF solution, resulting in three solutions (S1, S2, and S3). To optimize the dispersion of AgNPs within the polymer matrix, sonication was performed for 30 min at 25 Hz using a tip sonicator equipped with a 13 mm tip. Additionally, to mitigate potential thermal effects that could lead to polymer desorption and nanoparticle reaggregation, the samples were kept in an ice bath during sonication (see Scheme 1).

The aforementioned PAN/DMF and PAN/AgNP/DMF mixtures were electrospun into fiber mats using an EHD-TECH Lab-Research Electrospinning Machine. The electrospinning setup comprised a high-voltage power supply, a syringe pump, a spray tip with a 0.4 mm inner diameter, and a horizontal collector plate. The optimal electrospinning parameters, determined in previous studies [45], included a tip-to-collector distance of 13 cm, a flow rate of 0.8 mL/h, and onset voltages of 13 kV positive and 0.5 kV negative to achieve a stable Taylor cone. Then, the electrospinning process was carried out for 1 h in room conditions.

### 2.4. Characterization

The morphology and size of the synthesized structures were investigated using a field emission gun scanning electron microscope (FEG-SEM, Mira3 Tescan). Samples were directly deposited onto aluminum stubs and imaged at accelerating voltages ranging from 3 to 5 kV. Additionally, high-resolution Transmission Electron Microscopy (STEM) analysis was performed at 30 kV in bright field mode using the same FEG-SEM. X-ray diffraction (XRD) analysis was used to confirm the presence of AgNPs. XRD patterns were collected using an EMPYREAN diffractometer (PANalytical) in a Bragg–Brentano geometry with Cu K-α radiation (λ = 1.541 Å) at 45 kV and 40 mA. To minimize substrate interference, samples were mounted on stubs pre-coated with double-sided carbon tape and a 20 nm thin layer of conductive gold (99.99% purity) via a Quorum Q150R ES sputtering evaporator. Particle and fiber diameter distributions were statistically analyzed using ImageJ 1.54 software based on measurements of 82 individual fiber diameters. Sorption isotherms were generated using an Anton Parr Nova 600 instrument. The specific surface areas were determined within the range of *p*/*p*0 = 0.05–0.3 employing the BET method.

### 2.5. Evaluation of Catalytic Activity and Real-Time Electrochemical Sensing

Methyl orange (MO) was selected to evaluate the catalytic activity of the nanocomposite based on electrospun fiber mats functionalized with AgNPs. A membrane with dimensions of 2 cm × 2 cm and weight around 5 mg was submerged in a solution containing 15 mL of MO and sodium borohydride (NaBH_4_) solution (MO = 0.025 mM, NaBH_4_ = 2.5 mM), Subsequently, a 2 mL solution was extracted every time for the following decolorization and analyzed by UV-vis spectrophotometer. In addition to this conventional spectroscopic analysis, an electrochemical sensor platform was integrated to enable the real-time, in situ monitoring of the catalytic reaction. The active sensing layer, consisting of an electrospun membrane embedded with AgNPs, was electrically connected to both the working and counter electrodes. The reference electrode was directly immersed in the reaction solution. A microcontroller processed the digitized signals in real-time, streaming them to a Python-based (Phyton 3.12) graphical interface for data visualization and analysis (see Scheme 1).

### 2.6. Scalability and Reusability

Scalability was investigated by repeating the experiment with reaction volumes of 300 and 900 mL. To address potential membrane shrinkage observed during these larger-scale experiments, a custom-designed support structure fabricated via 3D printing with resin-based technology was utilized. Furthermore, the re-usability of the catalyst was investigated. After each reaction, the membrane was cleaned with water and dried for 1 h at 50 °C for further use.

## 3. Results and Discussion

### 3.1. Morphology and Structure of PAN/AgNPs Nanofiber Membranes

**Figure 1:**
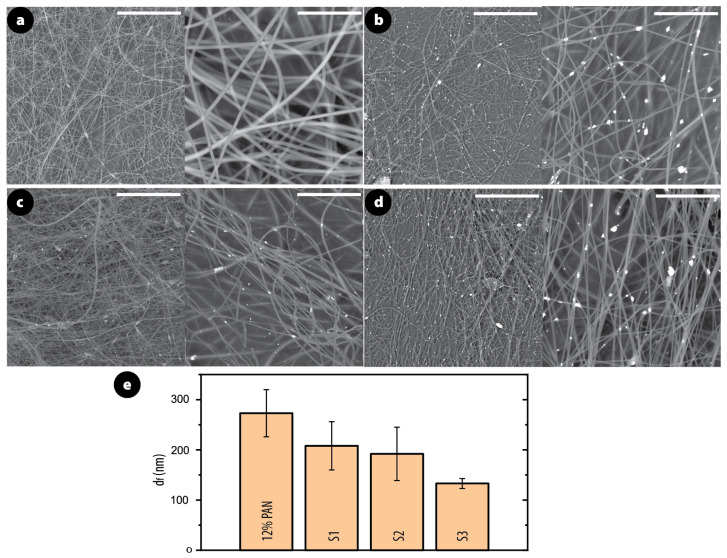
SEM images and fiber diameter $d_{f}$ distributions for 12% PAN with AgNPs concentrations of (**a**) 0%, (**b**) 2.5%, (**c**) 5%, and (**d**) 7.5%. Panel (**e**) summarizes the fiber diameter distributions. Scale bars represent 50 μm (left column) and 5 μm (right column).

**Figure 2:**
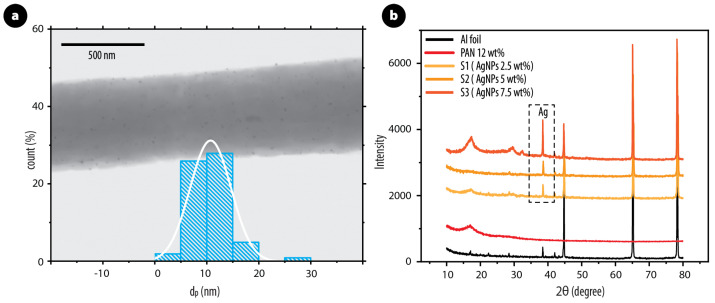
(**a**) SEM images of S2 and AgNP diameter $d_{p}$ distributions. (**b**) XRD patterns.

To elucidate the morphological characteristics of the fabricated nanocomposites, a combination of high-resolution microscopy techniques was employed: Scanning Electron Microscopy, Scanning Transmission Electron Microscopy, and Transmission Electron Microscopy. The resulting micrographs, presented in Figure 1 and Figure 2, offer a detailed visualization of the fiber structure and AgNPs distributed across the fiber surface, respectively. Additionally, Figure 1e illustrates the fiber diameter ($d_{f}$) distribution. PAN (12 wt.% + DMF) fibers without AgNPs were initially electrospun, as shown in Figure 1a. These fibers exhibited a smooth morphology with a well-defined structure. The average diameter, measured across 82 samples, was determined to be 273 nm ± 47 nm. In contrast, the incorporation of AgNPs, as depicted in Figure 1b–d, resulted in nanofibers with a smaller average diameter compared to PAN fibers without AgNPs. For instance, sample S1 (Figure 1b) with 2.5 wt.% AgNPs concentration had an average diameter ($d_{f}$) of 208 nm ± 48 nm. This trend continued with increasing AgNP content, with $d_{f}$ values of 192 nm ± 53 nm for S2 (Figure 1c) and a significantly reduced value of 133 nm ± 10 nm for S3 (Figure 1d). This diameter variation can be attributed to the presence and size of the NPs. As more AgNPs were incorporated, they released Ag^+^ ions into the working solution, enhancing its conductivity and viscosity. This effect was also observed in previous studies [46,47].

**Figure 3:**
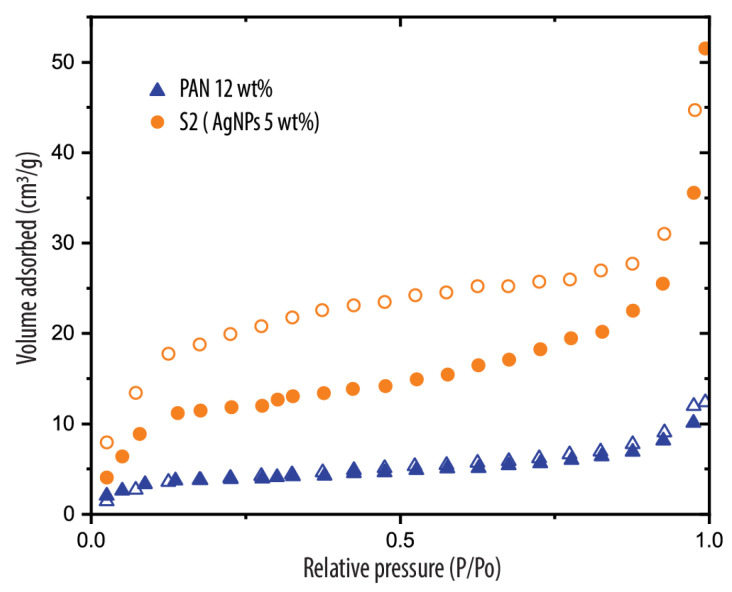
Surface area distribution curve. Hollow symbols indicate the desorption branch, whereas solid symbols correspond to adsorption.

Meanwhile, the AgNPs exhibited a generally dispersed morphology within the PAN nanofibers; as observed in the corresponding figures (Figure 1b–d), they demonstrated a more pronounced surface coverage, particularly on the fiber surface of S2 (see Figure 2a). The average diameter of these surface-bound AgNPs was meticulously measured from a representative section of a single fiber, given the high resolution required for accurate individual nanoparticle sizing. This yielded a particle diameter of $d_{p}$ = 10.73 nm ± 3.9 nm. This enhanced spatial arrangement aligns with observations from previous studies employing chemical synthesis or in situ assembly techniques for NP incorporation [26,48]. The Supplementary Material (Figure S1) shows the TEM micrographs of samples S1, S2, S3 and pure AgNPs. Finally, to further confirm the presence and distribution of AgNPs within the PAN nanofibers, X-ray diffraction was employed for both PAN and PAN/AgNPs composites. All samples were analyzed on aluminum (Al) foil, and the diffracted intensities were recorded between 10° and 80°. Figure 2b shows the XRD diffractograms of PAN and the PAN/AgNPs composite fibers. Note that the figure includes the patterns for Al foil. The literature reports the characteristic Bragg reflections for silver nanoparticles at 38.45°, 46.35°, 64.75°, and 78.05°, corresponding to the (1 1 1), (2 0 0), (2 2 0), and (3 1 1) planes, respectively [49,50,51]. As expected for the PAN/AgNPs nanocomposite fibers, the XRD pattern exhibits the combined peaks of the polymer and the Al substrate, along with the strongest AgNPs reflection at 38.45°. Notably, the intensity of this peak increases with increasing AgNP content, as shown in the inset of Figure 2b. Moreover, the N2 adsorption–desorption isotherms for PAN and PAN/AgNPs are depicted in Figure 3. Interestingly, incorporating silver nanoparticles significantly modifies the membrane structure, creating a much higher surface area for adsorption (from 12.6 to 40.3 m^2^/g). This comparison demonstrates how effectively silver nanoparticle incorporation enhances the adsorption properties of the PAN membrane, making it potentially much more effective for applications requiring high surface area or adsorption capacity, such as filtration, catalysis, or environmental remediation.

### 3.2. Catalytic Activity of PAN/AgNPs Nanofiber Membranes

**Figure 4:**
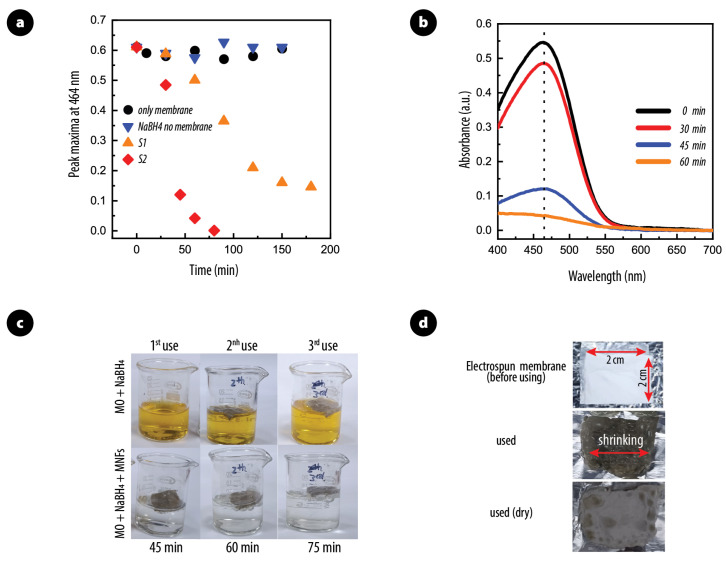
Optical study of methyl orange degradation: (**a**) UV–visible absorbance, (**b**) UV–visible spectra of S2 sample (dashed line indicates the absorption peak), (**c**) photoreaction time catalyzed, and (**d**) photographs of nanocomposite mats.

The reductive capacity of silver nanoparticles incorporated into polyacrylonitrile membranes was investigated for their potential role in azo dye reduction using NaBH_4_ as a reducing agent. First, it was observed that PAN/AgNP membranes did not absorb or degrade MO in the absence of a reducing agent (represented by black dots Figure 4a). Likewise, when NaBH_4_ was introduced without a membrane, no MO degradation occurred within the studied time frame (see blue inverted triangles). However, the presence of both components in the MO solution led to a significant dye reduction, as displayed by the yellow triangles and orange diamond for the S1 and S2 samples, respectively. As the results for S3 were identical to S2, these data were omitted. These findings suggest that the observed MO degradation is attributed to a catalytic mechanism involving MO, NaBH_4_, and the impregnated nanofiber membranes. On the other hand, to evaluate the reusability of the membranes and their catalytic activity after multiple uses, further experiments were conducted. However, extended reaction times were required for subsequent uses (45 min for the first use, 60 min for the second, and 75 min for the third), as shown in Figure 4c. This observed decrease in efficiency could be attributed to changes in the dimensions of the active area in the membrane after each reaction cycle, including the cleaning and drying steps. Figure 4d compares the membranes’ initial state to their morphology after undergoing these processes, suggesting potential shrinkage. Strategies to mitigate this issue will be elaborated upon in the subsequent section.

**Figure 5:**
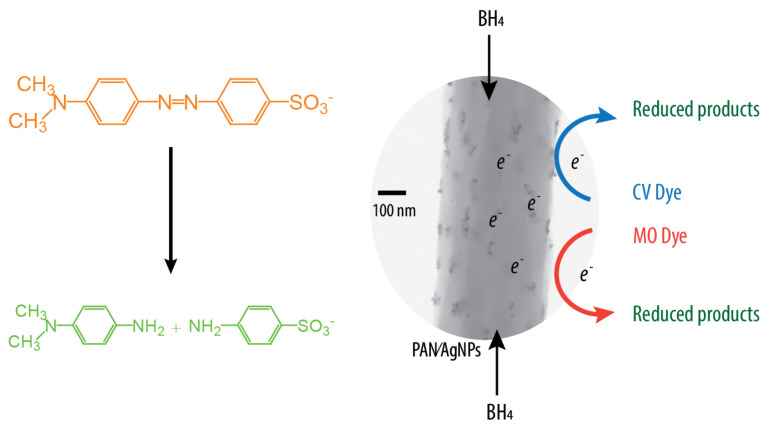
A scheme of the catalytic reduction mechanism.

Figure 4b shows that noticeable MO degradation occurs over time due to dye reduction. Membranes containing over 5 wt.% AgNPs achieved more than 95% degradation within 45 min (as seen in Figure 4b,c). This degradation is attributed to a catalytic process. NaBH_4_, acting as a reducing agent, leverages the silver nanoparticles’ surface as a platform to facilitate the conversion of azo groups (R-N=N-R) into colorless amines (-NH-NH-). As displayed in Figure 5 this mechanism involves three steps: (1) adsorption of both the dye and BH_4_- ions onto the silver nanoparticles; (2) electron transfer from BH_4_- to the dye, initiating the reduction; and (3) desorption of the transformed byproducts, allowing them to diffuse away from the catalyst.

*Monitoring chemical reaction:*

**Figure 6:**
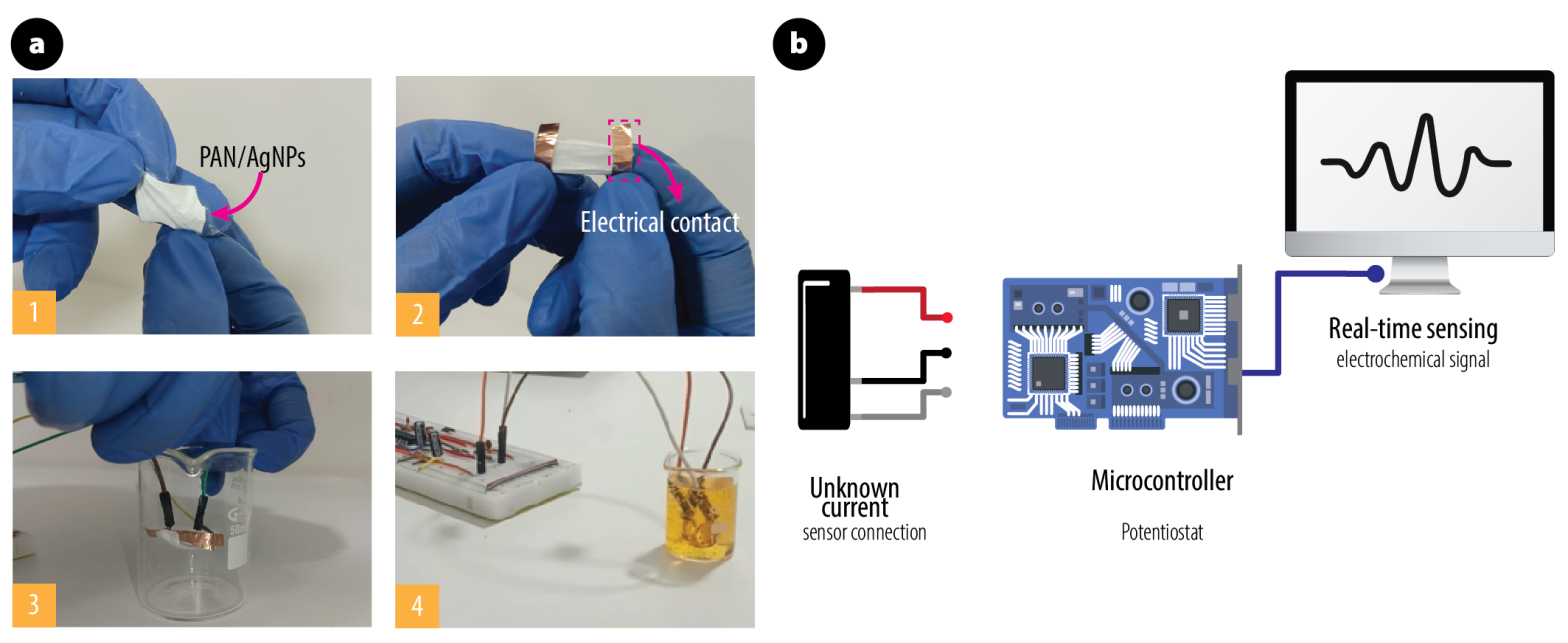
Schematic diagrams of the sensing platform. (**a**) Sensor evaluation in a reaction beaker setup: (**a-1**) PAN/AgNPs membrane, (**a-2**) copper tape electrodes, (**a-3**,**a-4**) immersed configuration. (**b**) Signal conditioning and acquisition via microcontroller.

Leveraging the well-documented semiconductor properties of silver nanoparticles and the electron transfer mechanism from BH_4_- to the dye, we performed a voltammetric analysis to monitor the chemical reactions. Subsequently, a three-electrode system was employed to assess the membrane’s conductivity during the reduction of methyl orange. This experimental configuration, illustrated in Figure 6, utilizes a microcontroller platform to maintain a constant potential between the working and reference electrodes via a potential controller. The potential controller can be adjusted to accommodate diverse experimental conditions [52].

**Figure 7:**
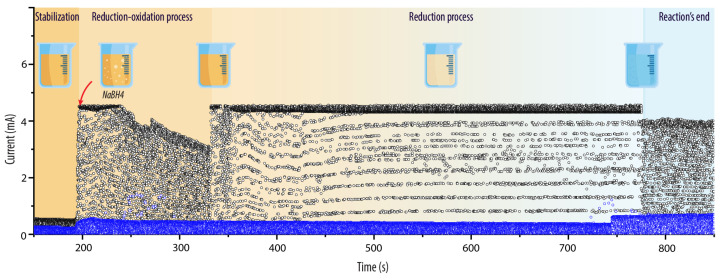
Sensed electrical behaviors of PAN (blue dots) and PAN/AgNP composites (black dots).

The performance of the sensor was evaluated in a reaction beaker setup (Figure 6a). A square PAN/AgNPs membrane (Figure 6a-1) was prepared with copper tape electrodes (Figure 6a-2) and immersed in the reaction setup (Figure 6a-3,a-4). The generated electrochemical current was amplified, converted to an electric signal by a conditioning circuit, and captured by a microcontroller (Figure 6b). This configuration enabled real-time monitoring and an in-depth analysis of current-time profiles through a Python-based software platform. Figure 7 illustrates the distinct electrical behaviors observed for both PAN and PAN/AgNP composites.

PAN membranes exhibited minimal electrical conductivity when immersed in the colored solution (Figure 7, blue dots). The introduction of NaBH_4_ did not significantly alter the conductivity, indicating a lack of significant electronic interaction between the membrane and the medium. This observation was corroborated by the absence of a discernible color change within the experimental timeframe. In contrast, PAN/AgNP composites displayed a markedly different response (Figure 7, black dots). Upon the addition of NaBH_4_, an immediate and substantial surge in conductivity was observed. This initial rise can reflect a competitive reduction–oxidation process occurring at the membrane–medium interface, where the silver nanoparticles act as active sites for electron transfer. Subsequently, a sustained increase in conductivity was evident, indicative of the ongoing reduction of the azo dye. This observation is corroborated by the disappearance of color within the measured timeframe, signifying the successful reduction of the azo dye. The sustained conductivity enhancement during this stage suggests the catalytic role of silver nanoparticles in facilitating electron transfer between the reducing agent and the dye molecule, thereby accelerating the reduction process. Once the dye molecules were completely reduced, the conductivity decreased, signaling the termination of the reaction.

*Scalability:*

**Figure 8:**
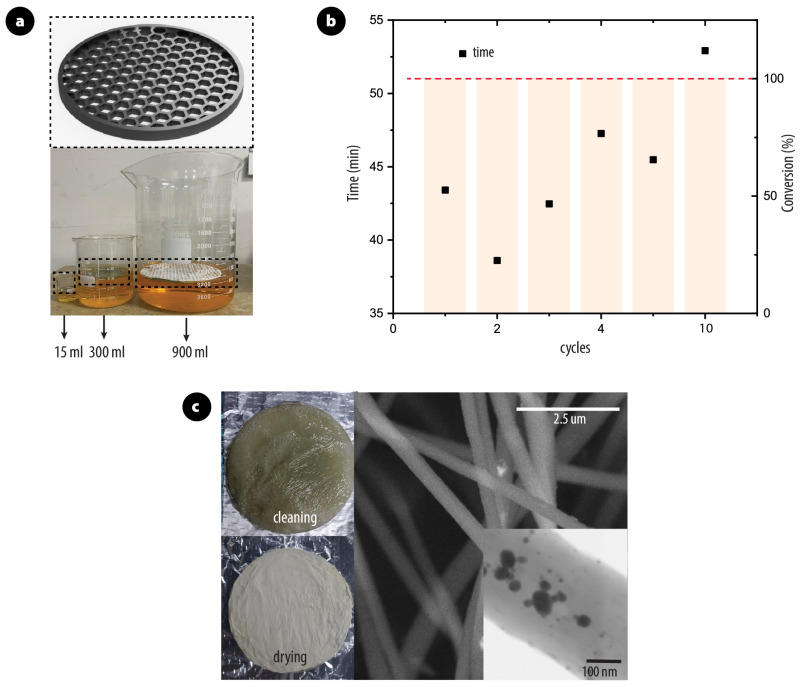
(**a**) Scalability of the catalytic and 3D-printed support structure (**top**). (**b**) Reusability evaluation: Catalytic performance after ten cycles. (**c**) Digital photograph of the membrane after cleaning and drying (**left**), and an SEM image of the same membrane following ten cycles of use (**right**).

Beyond catalytic efficiency, the practical implementation of catalysts depends on scalability and reusability. PAN/AgNPs nanocomposite have the advantage of easy removal from solutions after use, facilitating their reuse. To tackle the dual challenges of membrane shrinkage and limited scalability, we have engineered a novel 3D-printed support structure (see Figure 8a). This support provides robust mechanical stability to the membranes during the dye reduction process, allowing for a significant 60-fold increase in treatment volume from 15 mL to 900 mL while maintaining exceptional degradation performance.

Figure 8b illustrates the reusability of PAN/AgNPs membranes in degrading 300 mL of MO over ten cycles. Despite undergoing repeated use, the membranes consistently achieved 100% MO removal. Minor fluctuations in the degradation time were observed, with an average of 45 min per cycle, underscoring the membranes’ robust performance. It is important to note that a rigorous cleaning and drying protocol, as outlined in Supplementary Material (Video S1), was employed after each catalytic cycle.

As depicted in the SEM image in Figure 8c, the structure of the membrane remained pristine after ten cycles. The densely populated AgNPs showed no signs of detachment or agglomeration, affirming the nanoparticles’ robust immobilization within the PAN nanofiber matrix. This observation highlights the robust bonding of the AgNPs within the fiber structure, demonstrating that the cleaning and drying process does not affect catalytic activity.

## 4. Conclusions

The research centered on the development of PAN nanofibers incorporating AgNPs through the electrospinning technique, with a primary focus on refining AgNP concentration and dispersion within the fiber matrix. High-resolution microscopy provided detailed insights into the uniform dispersion of AgNPs at a 5 wt.% concentration across the PAN nanofiber surface. A notable reduction in fiber diameter, reaching a minimum of 133 nm ± 10 nm, was observed upon increasing the AgNP content to 7.5 wt.%. Complementary X-ray diffraction analysis substantiated the presence of AgNPs within the nanofibers, offering quantitative validation of successful nanoparticle integration.

The findings demonstrate that PAN/AgNP composite membranes exhibit exceptional potential for dye sensing and reduction. The marked increase in electrical conductivity upon NaBH_4_ introduction and the electrical conductivity decrease when the reduction process ends strongly suggest their suitability as dye reduction sensors. Moreover, these membranes displayed outstanding catalytic activity in the degradation of methyl orange using NaBH_4_, achieving optimum degradation rates for membranes containing more than 5 wt.% AgNPs. Additionally, the introduction of a novel 3D-printed support structure greatly enhanced the scalability of the PAN/AgNPs nanofiber membranes, allowing for a remarkable 60-fold increase in treatment volume without compromising degradation performance. The membranes also demonstrated exceptional reusability, consistently achieving 100% removal of methyl orange over ten consecutive cycles, with an average degradation time of 45 min per cycle. Importantly, the structural integrity of the membranes was maintained after repeated use, with no noticeable detachment or agglomeration of AgNPs, underscoring the robust durability of the nanofiber matrix.

## Data Availability

The original contributions presented in this study are included in the article/Supplementary Material. Further inquiries can be directed to the corresponding author.

## Supplementary Materials

The following supporting information can be downloaded at: https://www.mdpi.com/article/10.3390/polym17131762/s1, Figure S1: Typical TEM images of PAN/AgNP fibers showing embedded AgNPs. Images display: (a) S1 with 2.5 wt.% AgNPs, (b) S2 with 5 wt.% AgNPs, (c) S3 with 7.5 wt.% AgNPs, and (d) pure AgNPs; Video S1: Post-Reaction membrane cleaning procedure.
