# Supplementary material for: Polyacrylonitrile/Silver Nanoparticles Composite for Catalytic Dye Reduction and Real-Time Monitoring
Source: Polymers (Basel). 2025 Jun 26;17(13):1762. doi: 10.3390/polym17131762 (PMC12252141; doi:10.3390/polym17131762)
Supplement: Supplementary file 1 [file polymers-17-01762-s001.zip › Figure S1.pdf]

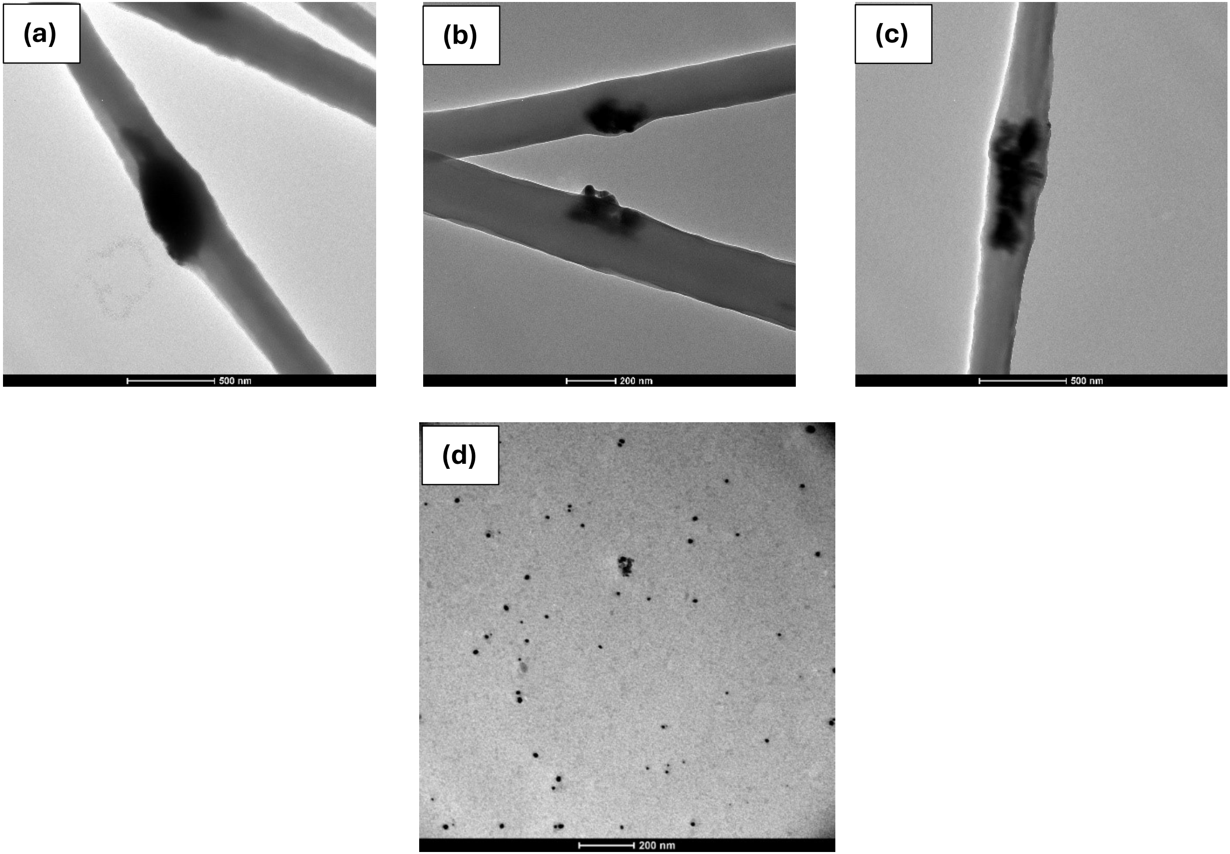

Figure S1. Typical TEM images of PAN/AgNP fibers showing embedded AgNPs. Images display: (a) S1 with 2.5 wt.% AgNPs, (b) S2 with 5 wt.% AgNPs, (c) S3 with 7.5 wt.% AgNPs, and (d) pure AgNPs.
